# Recruiting patients to a digital self-management study whilst in hospital for a chronic obstructive pulmonary disease exacerbation: A feasibility analysis

**DOI:** 10.1177/20552076211020876

**Published:** 2021-05-27

**Authors:** Maxine Whelan, Christopher Biggs, Carlos Areia, Elizabeth King, Beth Lawson, Nikki Newhouse, Xiaorong Ding, Carmelo Velardo, Mona Bafadhel, Lionel Tarassenko, Peter Watkinson, David Clifton, Andrew Farmer

**Affiliations:** 1 Nuffield Department of Primary Care Health Sciences, University of Oxford, Oxford, UK; 2 Centre for Intelligent Healthcare, Coventry University, Coventry, UK; 3 Nuffield Department of Clinical Neuroscience, University of Oxford, Oxford, UK; 4 Department of Engineering, Institute of Biomedical Engineering, University of Oxford, Oxford, UK; 5 Department of Respiratory Medicine, Nuffield Department of Medicine, University of Oxford, Oxford, UK; 6 Oxford-Suzhou Centre for Advanced Research, Suzhou, China

**Keywords:** Feasibility, respiratory, hospital admission, self monitoring, wearables, COPD

## Abstract

**Background:**

Patients with chronic obstructive pulmonary disease (COPD) are often hospitalised with acute exacerbations (AECOPD) and many patients get readmitted. Intervening with hospitalised patients may be optimal timing to provide support. Our previous work demonstrated use of a digital monitoring and self-management support tool in the community. However, we wanted to explore the feasibility of recruiting patients whilst hospitalised for an AECOPD, and to identify the rate of dropout attrition around admission for AECOPD.

**Methods:**

Patients were recruited to the EDGE2 study between May 2019 and March 2020. Patients were identified by the clinical teams and patients were recruited by members of the clinical research team. Participants were aged 40 years or older, had a diagnosis of COPD and were attending or admitted to hospital for an AECOPD. Participants were given a tablet computer, Bluetooth-linked pulse oximeter and wrist-worn physical activity monitor to use until 6 months post-discharge. Use of the system aimed to support COPD self-management by enabling self-monitoring of vital signs, COPD symptoms, mood and physical activity, and access to multi-media educational resources.

**Results:**

281 patients were identified and 126 approached. The main referral source was the specialist respiratory nursing and physiotherapist team (49.8% of patients identified). Twenty-six (37.1%) patients were recruited. As of 21 April 2020, 14 (53.8%) participants withdrew and 11 (of 14; 78.6%) participants withdrew within four weeks of discharge. The remaining participants withdrew between one and three months follow-up (1 of 14; 7.1%) and between three and six months follow-up (2 of 14; 14.3%).

**Conclusion:**

A large number of patients were screened to recruit a relatively small sample and a high rate of dropout was observed. It does not appear feasible to recruit patients with COPD to digital interventional studies from the hospital setting when they have the burden of coping with acute illness.

## Introduction

Patients with chronic obstructive pulmonary disease (COPD) are often hospitalised with acute exacerbations (AECOPD), presenting a mean age of 67 ± 8 years.^1^ This is a common cause of emergency admission to hospital^2^ and costs the NHS nearly £2 billion a year.^3^ Nearly half of patients are discharged within three days of admission^4^ but a quarter of patients are readmitted within 30 days of discharge.^5^ To further complicate COPD management, nearly three quarters of patients have additional comorbidities (including coronary artery disease and diabetes mellitus) and the prevalence of comorbid conditions increases with more advanced disease.^6^ This patient group has a high readmission rate, and in further testing the use of remote monitoring, our rationale for introducing the technology in a hospital setting is to minimise any delay in monitoring on return to the community. Prior to discharge, patients with COPD are administered a discharge care bundle as part of standard care (check inhaler technique and refer to appropriate services such as pulmonary rehabilitation and smoking cessation). Integrating a digital intervention into this existing care pathway could be an effective approach to prevent readmission with implementation as part of standard clinical pathways.

We have previously successfully evaluated a digital monitoring and self-management support tool with COPD patients in the community.^7^ Our evaluation reported low attrition (12.7%) during the 12-month study and 80% using the system daily.^8^ We have subsequently moved to identifying patients admitted with AECOPD to hospital to evaluate linkage of data from hospital and the support tool. This study summarises the feasibility of recruiting patients whilst hospitalised for an AECOPD (primary aim) and identifies the extent to which study participants withdraw (secondary aim).

## Methods

The sElf-management anD support proGrammE (EDGE2) for COPD research study was approved by the London-Surrey Research Ethics Committee (ref 18/LO/1939) in December 2018 and prospectively registered (ISRCTN82570166). Data reported here relates to the 11-month recruitment period between May 2019 and March 2020. Participants provided written informed consent.

Adults visiting or admitted to the John Radcliffe Hospital site of the Oxford University Hospitals NHS Foundation Trust (OUHT), were eligible if they were willing and able to give informed consent, aged 40 years or older, had a clinical diagnosis of COPD in their medical records, were hospitalised because of an exacerbation, were a current or ex-smoker, have a post-discharge destination that was not a medical facility or prison, lived in Oxfordshire or surrounding counties, were able to adequately understand verbal and written English, and were able to complete questionnaires and use a tablet computer. Patients were excluded if they had another substantial lung condition, had severe heart failure or had a life expectancy of less than six months. Clinical teams at the hospital were asked to identify potentially eligible patients whilst doing routine ward rounds. The clinical teams shared patients’ details with the clinical research team in the event the patient agreed they were happy to be approached about participating in research. The clinical research team then screened and enrolled patients.

Participant details were recorded in a screening and enrolment log, detailing the date, referral source (which clinical team member), whether they were subsequently approached and recruited, and reasons for not being approached, ineligibility and non-participation. Using the ICD-10 codes of J44.0, J44.1 and J44.9, the total number of patients at the hospital for an exacerbation during this period was extracted from routine electronic data recorded by the OUHT.

Participants were given a tablet computer containing the EDGE mHealth application, a Bluetooth-linked pulse oximeter and wrist-worn physical activity monitor. The devices were given to the participants whilst in hospital and participants were encouraged to use them up until six months post-discharge. Participants were given a verbal description of the system and a brief demonstration. Participants were encouraged to use the system to:

- Self-monitor vital signs (daily 60-second acquisition using pulse oximeter; heart rate and oxygen saturation parameters collected)
- COPD symptoms (daily questions via tablet computer; participant responses about cough, breathlessness and use of medications collected)
- Mood (monthly questions via tablet computer; participant responses to mood screening questionnaires [PHQ-2 and GAD-2] and subsequent PHQ-8 and/or GAD-7 collected where the screening questionnaires were positive)
- Physical activity (daily via physical activity monitor; step count collected).
- Access multi-media educational resources via the tablet computer to support their COPD self-management, such as videos to demonstrate correct inhaler technique, pulmonary rehabilitation exercises and self-management techniques for breathlessness.

The occurrence, timing and reasoning for participant withdrawals were recorded using REDCap electronic data capture tools hosted at the University of Oxford.^9^

## Results

### Recruitment

**Figure 1.**
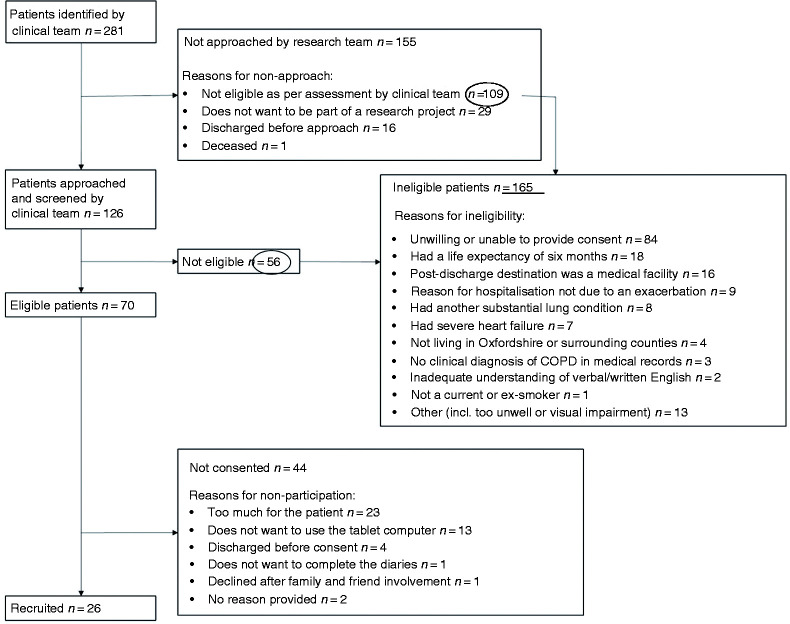
CONSORT flow of patients screened.

In total, 281 patients were identified by the clinical teams and 126 patients were approached (Figure 1). The clinical research team were most often informed about patients by specialist respiratory physiotherapists and nurses (n = 140, 49.8%) whilst remaining patients were referred by physiotherapists (n = 76, 27%), nurses (n = 56, 19.9%), doctors (n = 8, 2.8%) and other (n = 1, 0.3%). In total, 70 (55.5%) patients were eligible, of whom 26 (37.1%) patients were recruited. The main reason for ineligibility was patients lacking capacity (n = 84, 50.9%) and the main reason for non-participation was ‘too much going on’ (n = 23, 52.3%).

### Withdrawals

As of 21 April 2020, eight participants completed the study (of 26; 30.8%), four participants are still taking part and 14 (53.8%) participants have withdrawn. Six participants withdrew because they did not want to use the EDGE2 support tool and six participants withdrew because they did not feel well enough or found that they had “too much else going on” and the support tool was not manageable in parallel. Two participants died whilst in hospital (during the index admission and a re-admission).

The 14 participants who withdrew did so at varying points in the study. Eleven (of 14; 78.6%) participants withdrew before the one-month follow up, whilst one participant withdrew between one and three months follow-up (of 14; 7.1%) and two participants withdrew between three and six months after discharge (of 14; 14.3%).

## Discussion

Recruiting patients hospitalised for an AECOPD to the EDGE2 study evaluating use of a digital health intervention does not appear to be feasible. A large number of patients had to be screened to recruit a relatively small number of participants. The main concerns regarding the recruitment of hospitalised patients related to the burden of illness and capacity to engage with self-care after discharge, with a high rate of attrition also recorded.

A comparable digital intervention study recruiting patients hospitalised with AECOPD in the UK asked participants to wear a lower-back belt sitting monitor for two weeks after discharge. They also experienced low patient uptake (15.6% versus 37.1%) and a high proportion of participant withdrawals (48.5% versus 53.8%).^10^ Ineligibility reasons largely related to feeling too unwell (40%) and too severe comorbidities (36.4%). Common reasons for withdrawal related to feeling unwell and overwhelmed after experiencing an exacerbation (having lots of hospital appointments, readmissions, dealing with other comorbidities, medications and lack of social support). A trial of the MyCOPD app in patients recently hospitalised with COPD found that of the 124 potentially eligible patients, more than half declined study inclusion without giving a reason and recorded a 15% withdrawal rate.^11^ Another study enrolled patients with COPD whilst hospitalised but this time to a non-digital supported self-management programme (“SPACE For COPD).^12^ They revealed that 35.3% of approached patients declined participation in the study without reason, with others deciding not to participate due to comorbidities, mental health problems and being unable to read/see. In comparison to the present study, their withdrawal rate was much lower at 10.3%. A web-based version of that study (instead offering information on a tablet or PC) reported more than two thousand patients were screened to reach 100 participants (4.8% of patients screened). Predominate reasons for exclusion reported were not being web literate (69%), unwilling (15%) or had comorbidities precluding involvement (12%).^13^ Our study findings that recruiting patients with COPD during hospitalisation can be difficult appear to be supported by the literature.

Previous work in which the original EDGE system was evaluated with patients in the community demonstrated that patients were able to use the application, interpret clinical data, and use these within their self-management approach regardless of previous knowledge.^8^ The intervention development process focused on the provision of a simple and intuitive application that could be used - and which was used - regardless of participants’ previous computer experience or self-reported digital literacy. No changes have been made to the intervention components or delivery between that proof-of-concept study and the current work. It is therefore reasonable to assume that attrition in the current study can be explained more by implementation processes than by limitations in the intervention itself. The distinction between the introduction of an intervention in the community versus introduction during a hospital admission is likely to be a critical factor in the implementation process. Timing of introducing interventions around hospitalisation is important in this vulnerable patient group. It is possible that interventions that focus on non-intrusive monitoring by health care staff may be acceptable. But where patients need to make measurements or engage in self-management, the acute hospital may be a suboptimal setting to start a digital intervention.

Consideration needs to be given to the timing of introducing an intervention and balancing this against the burden of coping with acute illness. There is a risk that, if digital tools to support self-management were to be introduced into acute care settings, vulnerable patients could potentially be overburdened at a time when their illness was imposing great physical and psychosocial stress.
